# Hybrid PET/MRI in Inflammatory Cardiac Diseases: A Systematic Review and Single-Center Experience

**DOI:** 10.3390/diagnostics15131670

**Published:** 2025-06-30

**Authors:** Marco Fogante, Giulia Argalia, Paolo Esposto Pirani, Cinzia Romagnolo, Liliana Balardi, Giulio Argalia, Fabio Massimo Fringuelli, Nicolò Schicchi

**Affiliations:** 1 Maternal-Child-Senological-Cardiological Radiology and Outpatient Ultrasound, Department of Radiological Sciences, University Hospital of Marche, 60126 Ancona, Italy; paolo.espostopirani@ospedaliriuniti.marche.it (P.E.P.); giulio.argalia@ospedaliriuniti.marche.it (G.A.); nicolo.schicchi@ospedaliriuniti.marche.it (N.S.); 2 Nuclear Medicine, Department of Radiological Sciences, University Hospital of Marche, 60126 Ancona, Italy; giulia.argalia@ospedaliriuniti.marche.it (G.A.); cinzia.romagnolo@ospedaliriuniti.marche.it (C.R.); fabiomassimo.fringuelli@ospedaliriuniti.marche.it (F.M.F.); 3 Health Professions Area, Diagnostic Technical Area, University Hospital of Marche, 60126 Ancona, Italy; liliana.balardi@ospedaliriuniti.marche.it; 4 Cardiovascular Radiological Diagnostics, Department of Radiological Sciences, University Hospital of Marche, 60126 Ancona, Italy

**Keywords:** PET/MRI, hybrid imaging, inflammatory cardiac diseases, myocarditis, endocarditis, pericarditis, cardiac inflammation, multimodal imaging, diagnostic imaging, cardiac PET/MR

## Abstract

**Background/Objectives:** Hybrid positron emission tomography/magnetic resonance imaging (PET/MRI) is an emerging imaging modality that combines metabolic and anatomical data in a single session, offering promising diagnostic performance in various cardiac conditions. However, its role in inflammatory cardiac diseases (ICDs), such as myopericarditis and endocarditis, remains underexplored. This study aims to evaluate the diagnostic value of PET/MRI in ICDs through a systematic review of the literature, complemented by our institutional experience. **Methods:** We conducted a systematic review of PubMed, Scopus, and Embase up to April 2025, using keywords including “PET/MRI,” “PET/MR”, “myocarditis”, “endocarditis”, “pericarditis”, “inflammatory heart disease”, and “inflammatory cardiac disease”. Studies reporting the use of hybrid PET/MRI in ICDs were included and analyzed. Additionally, we retrospectively reviewed clinical and imaging data from 33 consecutive patients who underwent PET/MRI in our center for suspected myopericarditis or endocarditis between September 2022 and March 2025. **Results:** The systematic review identified 12 eligible studies evaluating PET/MRI in ICDs, highlighting its added value in cases with inconclusive findings on standalone MRI or PET. Common advantages reported included improved localization of active inflammation, better characterization of tissue damage, and enhanced diagnostic confidence. In our cohort, hybrid PET/MRI was considered diagnostically useful in 16/21 (76%) of myopericarditis cases and 9/12 (75%) of endocarditis cases, particularly for reclassifying uncertain findings and guiding clinical management. **Conclusions:** The combined analysis of the current literature and real-world clinical experience supports the diagnostic utility of hybrid PET/MRI in the evaluation of ICDs. This multimodal approach improves the interpretation of equivocal cases, facilitates accurate diagnosis, and may influence therapeutic decisions. However, larger prospective studies are needed to confirm these findings and establish standardized protocols.

## 1. Introduction

Hybrid positron emission tomography/magnetic resonance imaging (PET/MRI) is an advanced imaging modality that enables the simultaneous acquisition of metabolic, functional, and anatomical data [1,2,3]. By integrating the metabolic sensitivity of PET with the high spatial and tissue contrast resolution of MRI, this technology offers several advantages, including reduced radiation exposure, accurate spatial co-registration, and comprehensive disease assessment in a single session [4,5].

Initially limited by technical challenges such as mutual interference and MRI-based attenuation correction, PET/MRI has benefited from continuous technological refinements that have improved image quality, workflow efficiency, and clinical applicability [2,6]. These developments have expanded its use in various medical fields, particularly in cardiovascular imaging, where precise anatomical and metabolic characterization is essential in complex conditions such as inflammatory cardiac diseases (ICDs) [7,8]. ICDs encompass a spectrum of conditions characterized by inflammation of the myocardium, pericardium, or endocardium, most commonly manifesting as myopericarditis or infective endocarditis [9]. These disorders may result from infectious agents, such as bacteria or viruses, or from autoimmune or idiopathic mechanisms, leading to variable clinical presentations ranging from asymptomatic cases to severe heart failure, arrhythmias, or systemic embolism [10]. Timely and accurate diagnosis is often challenging due to the nonspecific nature of symptoms and overlapping imaging findings with other cardiac conditions. Conventional imaging modalities, such as echocardiography, may be inconclusive, especially in early or subclinical phases [11]. Cardiac MRI offers high-resolution tissue characterization and is particularly useful for detecting myocardial edema and fibrosis, while [18F]fluorodeoxyglucose(FDG)-PET enables visualization of metabolically active inflammatory foci [9,12]. However, when performed separately, these modalities may yield incomplete or ambiguous results [13]. The integration of these modalities through hybrid PET/MRI holds significant promise for improving diagnostic accuracy, allowing for a comprehensive assessment of both structural damage and active inflammation, which is critical for guiding clinical management and therapeutic decisions in patients with suspected ICDs [3,14].

Despite its potential benefits and growing interest, the application of PET/MRI in ICDs remains limited and poorly characterized. While a few narrative reviews have discussed the potential of PET/MRI in cardiovascular imaging, to our knowledge, no previous study has combined a systematic review specifically focused on ICDs with real-world clinical data. Moreover, existing reviews often address heterogeneous cardiovascular conditions, thereby limiting the depth of evaluation specific to ICDs [15,16,17,18,19,20,21,22,23,24].

In this context, combining a systematic review of published data with our institutional experience offers a broader and more reliable understanding of the diagnostic performance of PET/MRI in clinical practice. This dual approach enables the comparison and validation of findings from the literature against real-world data, helping to address current knowledge gaps and support the clinical integration of PET/MRI in the evaluation of ICDs.

Therefore, the aim of this study is to assess the diagnostic value of hybrid PET/MRI in patients with suspected ICDs by conducting a systematic review of the existing literature and integrating it with retrospective data from our single-center clinical experience.

## 2. Materials and Methods

### 2.1. Systematic Literature Review

The study protocol followed the Preferred Reporting Items for Systematic Reviews and Meta-Analyses (PRISMA-DTA) 2020 guidelines (PROSPERO ID: CRD420251064000).

A systematic literature review was conducted to evaluate the clinical utility of hybrid PET/MRI in the diagnosis of ICDs, including myocarditis, pericarditis, and infective endocarditis.

#### 2.1.1. Search Strategy

A comprehensive search was carried out in the PubMed, Scopus, and Embase databases up to 30 April 2025, using the following search string:

“pet/mri” OR “pet/mr” AND “(pericarditis OR myocarditis OR endocarditis OR inflammatory heart disease OR inflammatory cardiac disease)”

No date or language restrictions were applied. The reference lists of all the included articles were also manually screened to identify additional relevant studies.

#### 2.1.2. Inclusion Criteria

- Original clinical studies (prospective or retrospective), case series, or case reports reporting the use of hybrid PET/MRI in patients with suspected or confirmed ICDs.
- Studies that included imaging-based diagnostic assessment and provided qualitative or quantitative results (e.g., diagnostic yield, imaging findings, or clinical impact).
- Studies with clearly described patient cohorts and imaging protocols.

#### 2.1.3. Exclusion Criteria

- Reviews, editorials, conference abstracts without full text
- Studies using PET/CT or MRI alone
- Experimental animal studies or phantom studies
- Studies not focused on inflammatory cardiac disease
- Lack of specific data on inflammatory cardiac disease or mixed populations without separate analysis

#### 2.1.4. Study Selection and Data Extraction

**Chart 1:**
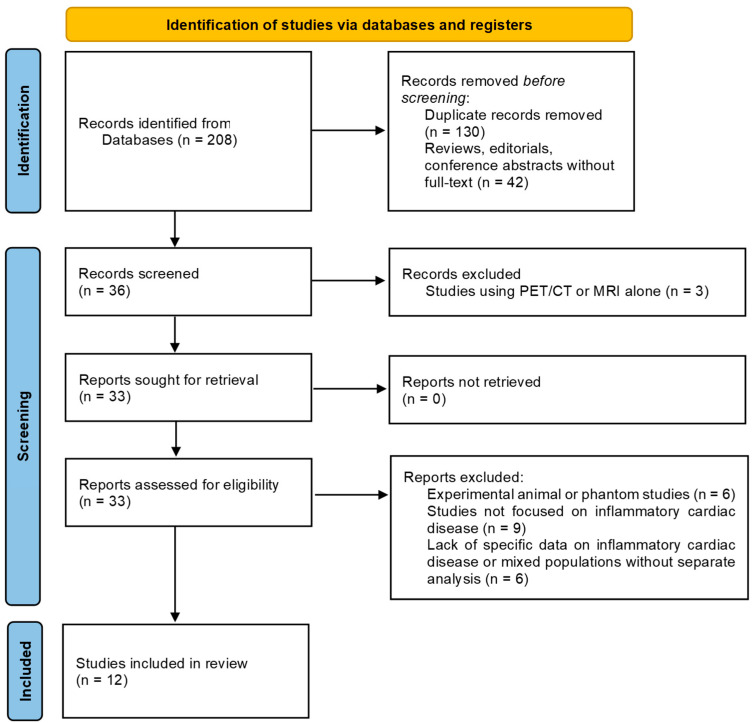
PRISMA 2020 flow diagram detailing the study selection process.

Two reviewers independently screened titles and abstracts for eligibility. Full texts of potentially relevant articles were reviewed in detail. Disagreements were resolved through discussion or consultation with a third reviewer. Data extracted from each study included: author, year, study design, number of patients, clinical indication, and main findings. Chart 1 shows a PRISMA flow diagram detailing the study selection process.

### 2.2. Institutional Experience

#### 2.2.1. Study Design and Population

This study was approved by the Institutional Review Board of Our Hospital (ID: 3584/Date of approval: 20 February 2025). In this retrospective observational study, we consecutively selected all patients older than 18 years who had undergone a [18F]FDG-PET/MRI examination, from the Radiological Database of our hospital, between September 2022 and March 2025. The inclusion criteria were: PET/MRI examination performed for suspected acute or chronic myopericarditis; PET/MRI examination performed for suspected endocarditis with an inconclusive diagnosis on echocardiography. The exclusion criteria were: absence of clinical data (*n* = 3) and non-diagnostic images (*n* = 1). Clinical data such as sex, age, weight, height, and cardiac symptoms were collected from all patients. The final study population consisted of 33 patients.

#### 2.2.2. Examination Protocol

All patients underwent a hybrid [18F]FDG-PET/MRI examination using a 3 Tesla PET/MRI Scanner (Signa, GE, Chicago, IL, USA), with 16-channel surface coils and cardiac synchronization and the same protocol. Seventy-two hours before the exam, all patients followed a carbohydrate-free, high-fat diet. The dose of [18F]FDG was calibrated to the patient’s weight and serum glucose levels were evaluated before [18F]FDG administration. PET/MRI data were acquired a median of 45–60 min after intravenous injection of [18F]FDG. A whole-body PET scan was performed (field of view: skull base—mid-thigh) with simultaneous acquisition of axial T1 and T2 weighted black-blood MRI sequences (approximate total duration 30 min). Next, a cardiac PET/MRI scan acquisition was carried out. The imaging procedure involved the simultaneous acquisition of [18F]FDG-PET and MRI images. The MRI sequences included T1-weighted imaging for anatomical assessment. Finally, cardiac-MRI acquisitions with coverage from the base to the apex of the heart with short-axis (SA), 2-chamber and 4-chamber cine sequences, SA black-blood T2-w sequences, and SA delayed enhancement sequences were acquired 10 min after gadobutrol (0.2 mmol/kg) injection.

#### 2.2.3. Image Analysis and Interpretation

All PET, MRI, and hybrid PET/MRI images were analyzed using dedicated software (Adw4.2, GE HealthCare, Chicago, IL, USA). Each imaging modality (MRI and PET) was independently evaluated by two experienced readers—one radiologist and one nuclear medicine physician—specialized in cardiovascular imaging. The MRI analysis included the automatic segmentation of epicardial and endocardial contours on short-axis views to calculate myocardial volumes, mass, and function using the Simpson method. The parameters assessed included end-diastolic volume (EDV), end-systolic volume (ESV), left ventricular ejection fraction (LVEF), and the presence and localization of myocardial edema and late gadolinium enhancement (LGE). The PET analysis focused on both the pattern and intensity of [18F]FDG uptake. The maximum standardized uptake value (SUVmax) was measured in focal areas of myocardial uptake, allowing for a semi-quantitative assessment of inflammatory activity. The diagnostic criteria for myopericarditis were defined as:

- MRI-positive: presence of both LGE and myocardial edema
- MRI-negative: absence of both LGE and edema
- MRI-inconclusive: LGE present without concomitant edema
- PET-positive: focal increased [18F]FDG uptake in the myocardium
- PET-negative: absence of abnormal [18F]FDG uptake
- PET-inconclusive: diffuse, mild, or anatomically uncertain [18F]FDG uptake

The diagnostic criteria for endocarditis were:

- MRI-positive: clear evidence of vegetations or periannular abscesses
- MRI-negative: normal valve morphology without signs of infection
- MRI-inconclusive: nonspecific findings (e.g., mild valve thickening or ambiguous enhancement) or technically limited assessment (e.g., prosthetic valves, arrhythmias)
- PET-positive: focal increased [18F]FDG uptake in the valvular region
- PET-negative: no abnormal [18F]FDG uptake
- PET-inconclusive: diffuse, low-grade, or anatomically unclear [18F]FDG uptake

Hybrid PET/MRI evaluation was subsequently performed in a multidisciplinary setting involving both imaging specialists. The integrated study was considered diagnostically useful when the combined morphologic, functional, and metabolic data, such as wall motion abnormalities, myocardial thinning, LGE, and focal [18F]FDG uptake, led to a clarified or refined diagnosis compared to either modality alone.

#### 2.2.4. Statistical Analysis

Statistical analysis was performed by the statistical software 14.8.1 MedCalc (MedCalc Software, Bvba, Oostende, Belgium). Descriptive statistics were used to summarize patient demographics, clinical characteristics, and imaging findings. Categorical variables were expressed as frequencies and percentages, while continuous variables were reported as mean ± standard deviation. The patients were divided into two groups, the first group with suspected myopericarditis and the second group with suspected endocarditis. The added diagnostic value of hybrid PET/MRI compared to individual modalities was evaluated based on the proportion of cases in which it provided a clarified diagnosis.

## 3. Results

### 3.1. Systematic Literature Review

Out of a total of 208 records identified, 24 articles were assessed for eligibility. Ultimately, 12 studies were included in the final qualitative synthesis, comprising eight prospective studies, three case series, and one case report. A PRISMA checklist and flow diagram detailing the study selection process is provided as Supplementary Materials. Across these studies, hybrid PET/MRI was performed in a total of 267 patients with suspected or confirmed ICDs. Overall, hybrid PET/MRI was reported to improve diagnostic evaluation in 10 studies and in 146 (54.7%) of the 267 patients included. Table 1 summarizes these results.

### 3.2. Institutional Experience

#### 3.2.1. Study Population

Of the 33 patients examined, 20 (61%) were male and 13 (39%) were female, with a mean age of 57.4 ± 14.2 years. Among the patients, 21 (64%) were referred for suspected myopericarditis and 12 (36%) for suspected endocarditis. Common clinical indications included chest pain, fever of unknown origin, elevated inflammatory markers, and cardiac dysfunction. The average [18F]FDG dose administered was 280.2 ± 30.5 MBq. Table 2 reports the clinical findings for both subgroups.

#### 3.2.2. Images Analysis and Interpretation

In the myopericarditis group (*n* = 21), patients showed a mean EDV index of 92 ± 18 mL/m^2^, ESV index of 45 ± 12 mL/m^2^, and LVEF of 55 ± 8%. LGE was present in 81% of patients. PET imaging revealed low metabolic activity with a mean SUVmax of 2.9 ± 1.7. In the endocarditis group (*n* = 12), EDV and ESV indexes were slightly lower (85 ± 20 and 40 ± 15 mL/m^2^), with an average LVEF of 53 ± 10%. LGE was detected in 25% of patients. PET imaging revealed higher metabolic activity with a mean SUVmax of 4.7 ± 1.3. Table 3 summarizes these results.

Among the 21 patients with suspected myopericarditis, MRI findings were positive for active inflammation in one patient (5%), negative in four patients (19%), and inconclusive in 16 patients (76%). [18F]FDG-PET was negative in 90% of cases and inconclusive in 10%, with no definite positive findings. However, hybrid PET/MRI led to diagnostic reclassification in 76% of cases. Among the 16 MRI-inconclusive cases, PET/MRI was able to reclassify 15 (93.8%) as negative due to the absence of metabolic uptake, effectively excluding active inflammation. One patient (6.2%) remained inconclusive despite combined imaging. The only patient with a positive MRI result had an inconclusive PET (for uncertain anatomical localization of the [18F]FDG uptake area), and the diagnosis of pericarditis was established based on the anatomical localization of the enhancement on MRI.

In the 12 patients with suspected endocarditis, MRI was inconclusive in most cases (10/12, 83.3%), with only one positive and one negative result. [18F]FDG-PET was positive in four patients (33.3%), negative in seven (58.3%), and inconclusive in one case (8.3%). Among the 10 patients with inconclusive MRI findings, hybrid PET/MRI enabled diagnostic reclassification in nine cases (90%): three were confirmed as positive due to focal PET uptake consistent with active infection, and six were reclassified as negative in the absence of [18F]FDG uptake. Only one case remained inconclusive despite combined imaging. Overall, PET/MRI provided significant diagnostic clarification in 75% of all suspected endocarditis cases, underscoring its added value, particularly in scenarios where MRI alone is non-diagnostic.

**Chart 2:**
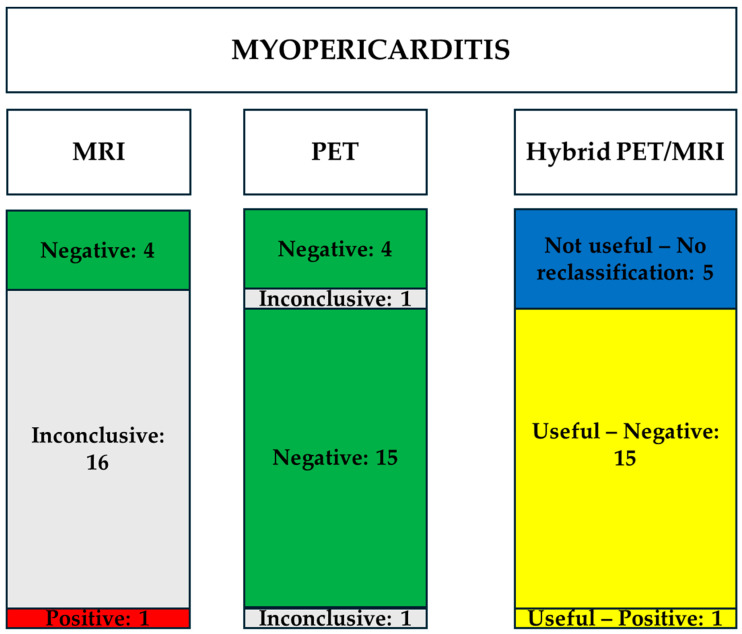
Illustration of diagnostic contribution of MRI, [18F]FDG-PET, and hybrid PET/MRI in patients with suspected myopericarditis. Abbreviations—MRI: magnetic resonance imaging; PET: positron emission tomography.

**Chart 3:**
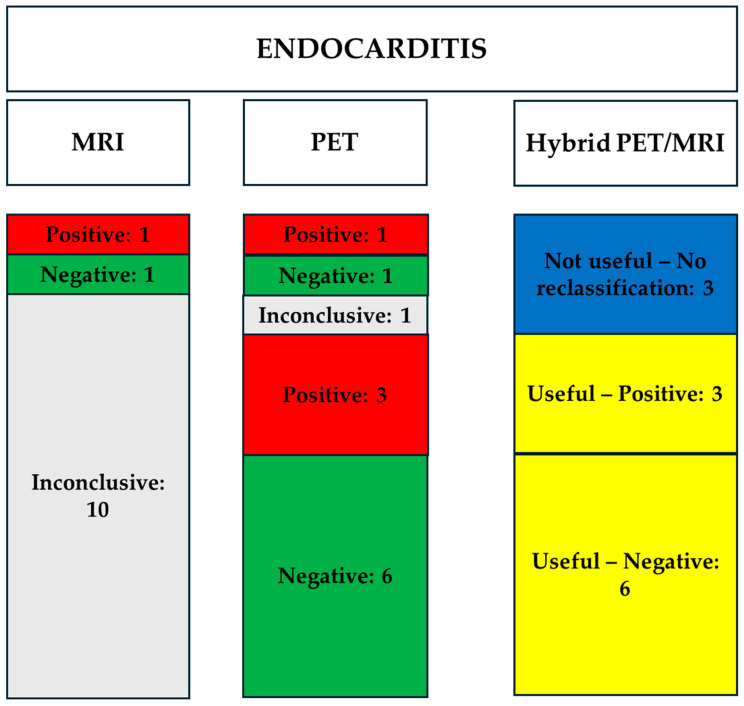
Illustration of diagnostic contribution of MRI, [18F]FDG-PET, and hybrid PET/MRI in patients with suspected endocarditis. Abbreviations—MRI: magnetic resonance imaging; PET: positron emission tomography.

Table 4 presents an overview of these results, while Table 5 and Table 6 summarize the diagnostic contribution of MRI, [18F]FDG-PET, and hybrid PET/MRI in patients with suspected myopericarditis and endocarditis, respectively. Chart 2 and Chart 3 have been included to visually illustrate the data from Table 5 and Table 6.

**Figure 1:**
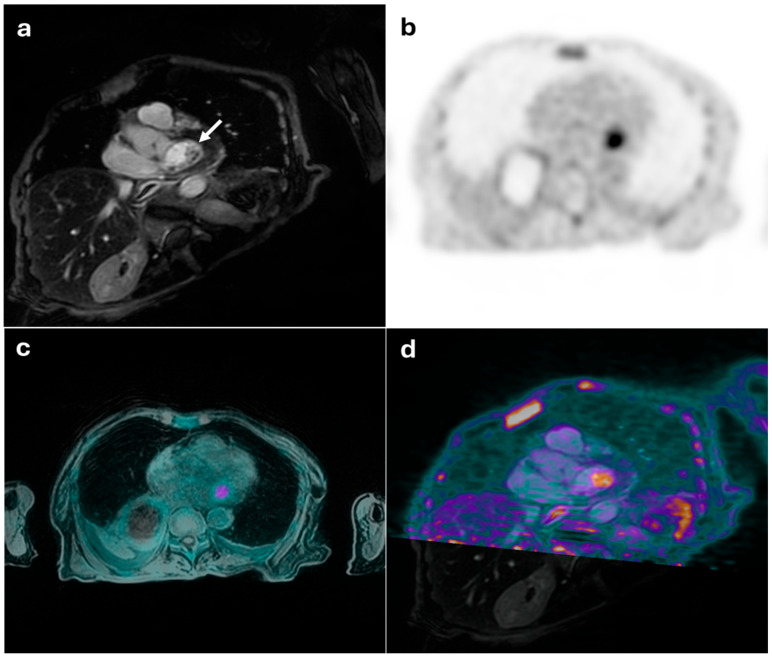
**45-year-old female with suspected mitral valve endocarditis.** (**a**) shows a cardiac MRI image revealing a mobile pseudonodular lesion on the posterolateral leaflet of the mitral valve, exhibiting late gadolinium enhancement (white arrow), which was inconclusive for the presence of inflammatory vegetation. (**b**) displays an FDG-PET image with pathological radiotracer uptake (SUVmax: 6.5). The integrated PET/MRI findings were diagnostic of mitral valve endocarditis (**c**,**d**).

**Figure 2:**
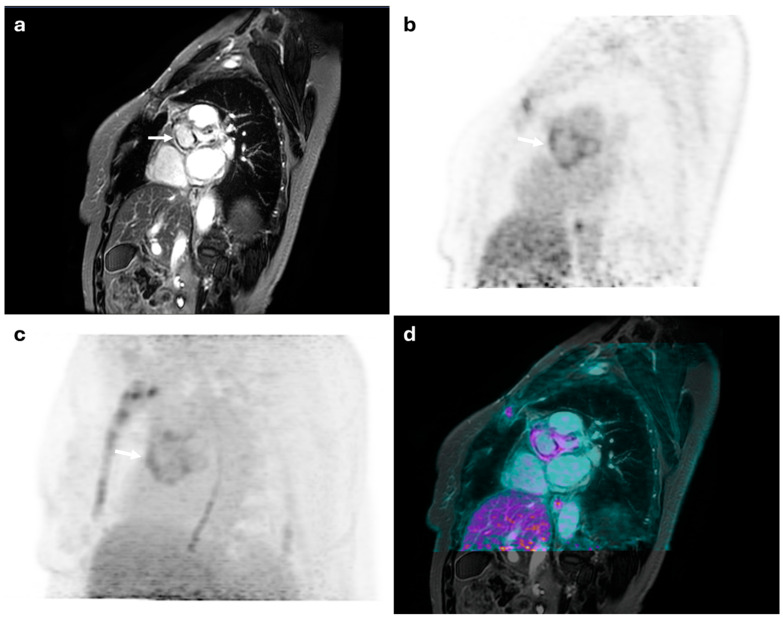
**52-year-old female with suspected aortic valve endocarditis.** (**a**) shows a cardiac MRI image revealing thickening at the level of the aortic root in the paravalvular region, with extension into the ascending thoracic aorta. Late gadolinium enhancement highlights linear intramyocardial fibrosis in the paravalvular aortic area (white arrow). (**b**,**c**) display FDG-PET images demonstrating diffuse increased metabolic radiotracer uptake (SUVmax: 5 and 8) (white arrows). The combined PET/MRI findings were diagnostic of mitral valve endocarditis with extension to the sternum—predominantly the manubrium—and the adjacent retrosternal soft tissues at the same level (**d**).

**Figure 3:**
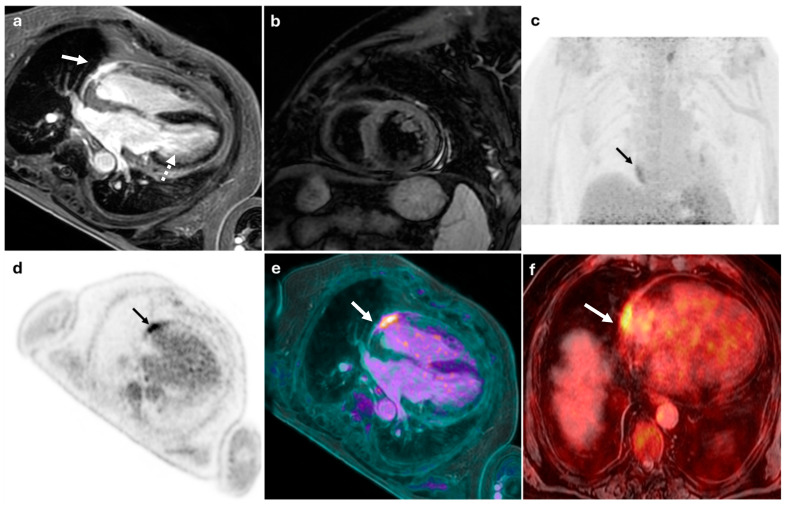
**56-year-old male with suspected myopericarditis.** (**a**) displays an MRI image showing epicardial late gadolinium enhancement localized to the mid-basal inferolateral wall of the left ventricle (white dashed arrow) and the anterolateral wall of the right ventricle (white arrow), without evidence of myocardial edema (**b**). (**c**,**d**) show FDG-PET images with pathological focal glucose hyperaccumulation in the pericardium at the anterolateral aspect of the heart (SUVmax: 5), although precise anatomical localization was inconclusive (black arrows). The combined PET/MRI findings were conclusive for right ventricular myopericarditis (white arrows) (**e**,**f**).

**Figure 4:**
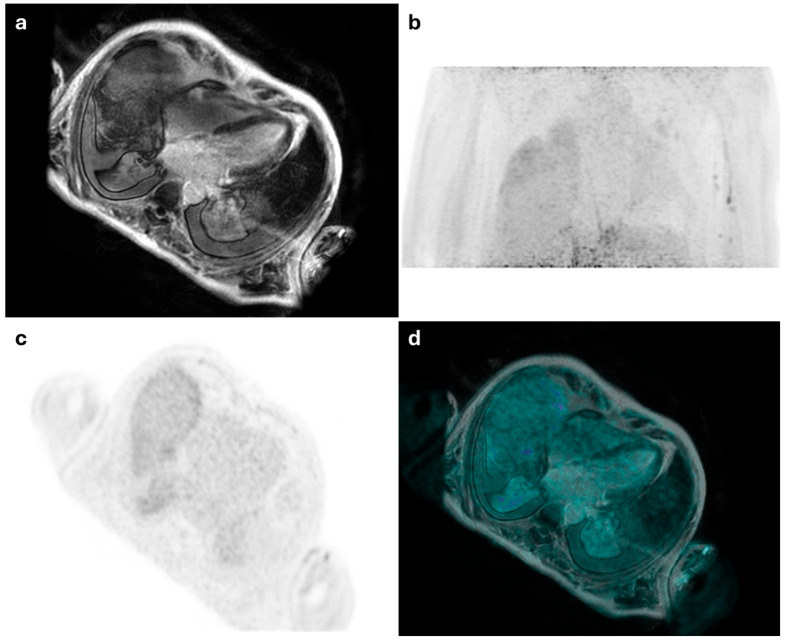
**55-year-old male with suspected myopericarditis.** (**a**) displays an MRI image showing a linear myo-epicardial late gadolinium enhancement in the mid-basal inferolateral wall of the left ventricle, suggestive of non-ischemic pattern fibrosis, possibly indicative of prior myocarditis. (**b**,**c**) show PET images with no distinct areas of radiotracer uptake in the myocardial walls, consistent with a negative result. The combined PET/MRI findings were conclusive for non-active myocarditis of the left ventricle (**d**).

Figure 1, Figure 2, Figure 3 and Figure 4 illustrate four case examples from our hospital experience.

## 4. Discussion

Recent technological advances have enabled the development of hybrid PET/MRI, allowing for the simultaneous acquisition of metabolic, anatomical, and functional information in a single imaging session [37]. This approach has shown particular promise in cardiovascular imaging by combining the high sensitivity of PET for detecting active inflammation with the superior tissue characterization and spatial resolution of MRI [38,39,40]. However, its application in ICDs remains limited. Based on this context, this paper aims to evaluate the diagnostic value of PET/MRI in ICDs through a systematic review of the literature, complemented by our institutional experience.

The 12 studies included in our systematic review enrolled a total of 267 patients with suspected or confirmed ICDs. Although methodologies varied, 10 out of 12 studies explicitly reported that hybrid PET/MRI provided a diagnostic benefit. Aggregated across these studies, 146 of 267 patients (54.7%) were reported as having improved diagnostic accuracy with PET/MRI. Although the number of published clinical studies remains limited, the available evidence consistently highlights the diagnostic value of hybrid imaging compared to standalone MRI or [18F]FDG-PET, particularly in challenging or inconclusive clinical scenarios. For example, Wisenberg et al. [28] and Greulich S et al. [33] demonstrated the utility of PET/MRI in detecting ICDs in cardiac sarcoidosis, while Hanneman et al. [26] confirmed its feasibility in both sarcoidosis and myocarditis. In 55 patients with myocarditis, Nensa et al. [27] demonstrated a good correlation between the sites of inflammation detected by [18F]FDG-PET and those identified by LGE and T2-weighted sequences on cardiac MRI. Moreover, they highlighted the potential of [18F]FDG-PET to detect extracardiac inflammatory involvement. Chau et al. [34] and Marschner et al. [35] highlighted the sensitivity of PET/MRI in detecting inflammatory sequelae following radiotherapy and COVID-19 vaccination, respectively, a finding also observed in a single case report by Lee et al. [32]. In smaller cohorts, such as those presented by Palmisano et al. [29] and Chen et al. [31], PET/MRI enabled differentiation between active inflammation and scar tissue in patients with persistent arrhythmias or myocarditis associated with immunotherapy. The study by Barrio et al. [30] was one of the few to investigate both myocarditis and endocarditis, confirming the added value of PET/MRI in identifying active disease components not visible on MRI alone. Finally, Trivieri et al. [36] investigated post-COVID-19 myocardial inflammation, demonstrating the sensitivity of PET/MRI in detecting subclinical inflammation.

Common technical protocols included T2-weighted and LGE sequences for MRI, combined with FDG-PET after myocardial suppression using a fat-rich diet. The systematic review highlighted several limitations across the available studies. Most were small, single-center, and with heterogeneous imaging protocols and patient preparation strategies. The importance of strict adherence to preparation protocols and careful image interpretation was emphasized by Abgral et al. [25], who reported a false-positive case due to inadequate suppression of physiological myocardial [18F]FDG uptake, despite dietary preparation. Overall, the included studies emphasized four recurring advantages: improved diagnostic confidence in ambiguous cases; better anatomical localization of [18F]FDG uptake; the ability to distinguish active from chronic inflammatory processes, thereby enhancing clinical decision-making; and the possibility of detecting extracardiac sites of inflammation.

Our institutional experience aligned with these findings: PET/MRI hybrid imaging led to a clarified or reclassified diagnosis in 76% of myopericarditis and 75% of endocarditis cases. This underscores the consistency and reliability of PET/MRI across different clinical settings. In myocarditis, MRI may show LGE without concurrent edema, leading to uncertainty about disease activity. In patients with suspected myocarditis, several studies reported that MRI findings, particularly LGE, can be nonspecific or reflect chronic, non-active disease. PET alone, while capable of detecting active inflammation, may lack anatomical precision. Hybrid PET/MRI overcomes these limitations by simultaneously integrating functional, metabolic, and structural data, which enables better differentiation between active vs. chronic inflammation, a critical distinction for guiding immunosuppressive therapy or clinical follow-up. In endocarditis, hybrid PET/MRI has shown utility in patients with prosthetic valves or equivocal echocardiographic findings. PET detects metabolically active infectious foci, while MRI delineates valve and perivalvular anatomy. Our own findings confirm this synergy, particularly in reclassifying cases with subtle or ambiguous imaging features. Nevertheless, our experience and the literature support the growing role of PET/MRI in the diagnostic workflow of ICDs. Its ability to integrate metabolic and anatomical data in a single session offers distinct clinical advantages, especially when conventional imaging is inconclusive. As technology evolves and protocols are standardized, PET/MRI may become increasingly incorporated into routine clinical practice for selected patients. Our results align with the limited existing data, such as the study by Barrio et al. [30], which also demonstrated the superiority of hybrid PET/MRI in the detection of myopericarditis and endocarditis.

Alongside these advantages, however, there are also several limitations that currently hinder the widespread adoption and routine clinical use of PET/MRI. Hybrid PET/MRI faces several technical and logistical challenges that currently limit its widespread adoption. These include the need for specialized equipment, prolonged scan times, complex MRI-based attenuation correction—which may affect PET quantification—and rigorous patient preparation protocols. As a result, its clinical use remains largely confined to specialized centers. Importantly, cost-effectiveness and outcome-driven studies are still lacking but will be essential to support the integration of PET/MRI into routine clinical practice.

The rationale for combining a systematic literature review with our single-center experience lies in the need to strengthen the current evidence base on PET/MRI in ICDs. While the reviewed studies suggest promising diagnostic utility, they often suffer from small sample sizes, variable protocols, and limited reproducibility. By integrating our clinical data, we provide confirmatory evidence in a real-world setting, reinforce the diagnostic value of hybrid PET/MRI across diverse scenarios, and contribute to bridging the gap between research and routine clinical applications, offering a more comprehensive and clinically grounded evaluation of the diagnostic role of hybrid PET/MRI in ICDs.

Future research should focus on the development and validation of diagnostic criteria and standardized algorithms that incorporate PET/MRI into the clinical management of ICDs. These efforts are crucial to support broader clinical use and ensure consistent interpretation across centers. In addition, the design of novel PET tracers, particularly those targeting macrophages or specific inflammatory pathways, offers promising avenues to improve diagnostic specificity and deepen our understanding of underlying disease mechanisms [39]. Further prospective studies are also needed to determine the prognostic value of hybrid PET/MRI in assessing treatment response and predicting long-term outcomes, potentially strengthening its role in clinical decision-making.

## 5. Conclusions

Hybrid PET/MRI represents a transformative tool in the management of ICDs. By combining metabolic and morpho-functional data, it overcomes the limitations of standalone PET or MRI, enabling more accurate diagnoses and personalized treatment strategies. While challenges remain, the clinical benefits justify its growing adoption in specialized centers. Future advancements in technology and larger-scale studies will further solidify its role in cardiovascular imaging.

## Figures and Tables

**Table 1:** Summary of Studies Reporting Hybrid PET/MRI in Inflammatory Cardiac Diseases.

Author—Year	Study Design	Number of Patients	Clinical Indication	Main Findings	Patients in Whom PET/MRI Added Diagnostic Value (*n*, %)
Abgral R et al., 2017 [25]	Case series	3	ICDs after myocardial injury	PET/MRI was useful for the detection and localization of ICDs	3 (100%)
Hanneman K et al., 2017 [26]	P	10	ICDs in myocarditis and sarcoidosis	PET/MRI was feasible in the detection of ICDs in cardiac sarcoidosis and myocarditis	0 (0%)
Nensa F et al., 2018 [27]	P	55	Myocarditis	[18F]FDG-PET was in good agreement with CMR findings	0 (0%)
Wisenberg G et al., 2020 [28]	P	10	ICDs in sarcoidosis	PET/MRI provided further insights into the disease process	10 (100%)
Palmisano A et al., 2021 [29]	Case series	2	Myocarditis in patients with persisting arrhythmias	PET/MRI detected active inflammation for scar in patients with persisting arrhythmias after myocarditis	2 (100%)
Barrio P et al., 2021 [30]	P	10	Myocarditis and endocarditis	PET/MRI was useful and provided additional data	7 (70%)
Chen Y et al., 2021 [31]	Case reports	2	Myocarditis in immunotherapy	PET/MRI was useful for the detection of ICDs	2 (100%)
Lee CH et al., 2022 [32]	Case report	1	Myocarditis after a COVID-19 mRNA vaccination	PET/MRI clearly visualized focal myocarditis	1 (100%)
Greulich S et al., 2022 [33]	P	43	ICDs in sarcoidosis	PET/MRI was useful for the detection of ICDs in cardiac sarcoidosis	33 (76.7%)
Chau OW et al., 2023 [34]	P	15	ICDs after radiotherapy	PET/MRI was sensitive to detecting acute cardiac inflammatory response to RT	15 (100%)
Marschner CA et al., 2023 [35]	P	17	ICDs after COVID-19 vaccination	PET/MRI showed evidence of myocardial inflammation after COVID-19 vaccination	17 (100%)
Trivieri MG et al., 2025 [36]	P	99	ICDs after COVID-19	PET/MRI was useful for the detection of ICDs	56 (56.6%)

Abbreviations—P: prospective; ICD: inflammatory cardiac disease; PET/MRI: positron emission tomography/magnetic resonance imaging; COVID-19: coronavirus disease 2019.

**Table 2:** Clinical Characteristics of Patients with Suspected Myopericarditis and Endocarditis.

Variable	Total (*n* = 33)
Age (years, mean ± SD)	57.4 ± 14.2
Sex (M/F, *n* [%])	20 (61%)/13 (39%)
Weight (kg, mean ± SD)	75.6 ± 12.5
Height (cm, mean ± SD)	170 ± 9
Clinical presentations	Chest pain Fever of unknown origin Elevated inflammatory markers Cardiac dysfunction
18F-FDG dose (MBq, mean ± SD)	280.2 ± 30.5
Radiation dose (mSv, mean ± SD)	5.6 ± 1.2

Abbreviations—SD: standard deviation; M: male; F: female; FDG: fluorodeoxyglucose.

**Table 3:** Quantitative MRI and PET Imaging Parameters in Patients with Suspected Myopericarditis and Endocarditis.

Parameter	Myo-Pericarditis (*n* = 21)	Endocarditis (*n* = 12)
**MR Data**		
EDV Index (mL/m^2^)	92 ± 18	85 ± 20
ESV Index (mL/m^2^)	45 ± 12	40 ± 15
LVEF (%)	55 ± 8	53 ± 10
Edema	1 (5%)	0 (%)
Late Gadolinium Enhancement, *n* (%)	17 (81%)	3 (25%)
**PET Data**		
SUVmax	2.9 ± 1.7	4.7 ± 1.3

Abbreviations—EDV: end-diastolic volume; ESV: end-systolic volume; LVEF: left ventricle ejection fraction.

**Table 4:** Overview of diagnostic contribution of Hybrid PET/MRI in Suspected Myopericarditis and Endocarditis.

Modality	Myopericarditis (*n* = 21)	Endocarditis (*n* = 12)
MRI		
Positive	1 (5%)	1 (8%)
Negative, *n* (%)	4 (19%)	1 (8%)
Inconclusive, *n* (%)	16 (76%)	10 (84%)
PET		
Positive	0 (0%)	4 (33%)
Negative, *n* (%)	19 (90%)	7 (58%)
Inconclusive, *n* (%)	2 (10%)	1 (9%)
Hybrid PET/MRI Useful	16/21 (76%)	9/12 (75%)

Abbreviations—MRI: magnetic resonance imaging; PET: positron emission tomography.

**Table 5:** Diagnostic contribution of MRI, [18F]FDG-PET, and hybrid PET/MRI in patients with suspected myopericarditis.

Patients with Suspected Myopericarditis				
	MRI Results	PET Results	Hybrid PET/MRI Diagnostic Reclassification of the Inconclusive Cases	What Helped the Reclassification of Inconclusive Cases
**1**	Inconclusive	Negative	Useful—Negative	The lack of uptake in PET excludes the presence of active inflammation
**2**	Inconclusive	Negative	Useful—Negative	The lack of uptake in PET excludes the presence of active inflammation
**3**	Inconclusive	Negative	Useful—Negative	The lack of uptake in PET excludes the presence of active inflammation
**4**	Negative	Negative	Not useful—No reclassification	
**5**	Inconclusive	Negative	Useful—Negative	The lack of uptake in PET excludes the presence of active inflammation
**6**	Inconclusive	Negative	Useful—Negative	The lack of uptake in PET excludes the presence of active inflammation
**7**	Inconclusive	Negative	Useful—Negative	The lack of uptake in PET excludes the presence of active inflammation
**8**	Negative	Negative	Not useful—No reclassification	
**9**	Inconclusive	Negative	Useful—Negative	The lack of uptake in PET excludes the presence of active inflammation
**10**	Inconclusive	Inconclusive	Not useful—No reclassification	
**11**	Inconclusive	Negative	Useful—Negative	The lack of uptake in PET excludes the presence of active inflammation
**12**	Inconclusive	Negative	Useful—Negative	The lack of uptake in PET excludes the presence of active inflammation
**13**	Negative	Negative	Not useful—No reclassification	
**14**	Positive	Inconclusive	Useful—Positive	MRI anatomically localizes the uptake in the pericardium, making the examination positive for pericarditis
**15**	Inconclusive	Negative	Useful—Negative	The lack of uptake in PET excludes the presence of active inflammation
**16**	Inconclusive	Negative	Useful—Negative	The lack of uptake in PET excludes the presence of active inflammation
**17**	Inconclusive	Negative	Useful—Negative	The lack of uptake in PET excludes the presence of active inflammation
**18**	Inconclusive	Negative	Useful—Negative	The lack of uptake in PET excludes the presence of active inflammation
**19**	Inconclusive	Negative	Useful—Negative	The lack of uptake in PET excludes the presence of active inflammation
**20**	Negative	Negative	Not useful—No reclassification	
**21**	Inconclusive	Negative	Negative	The lack of uptake in PET excludes the presence of active inflammation

Abbreviations—MRI: magnetic resonance imaging; PET: positron emission tomography.

**Table 6:** Diagnostic contribution of MRI, [18F]FDG-PET, and hybrid PET/MRI in patients with suspected endocarditis.

Patients with Suspected Endocarditis				
	MRI Results	PET Results	Hybrid PET/MRI Diagnostic Reclassification of the Inconclusive Cases	What Helped the Reclassification of Inconclusive Cases
**1**	Inconclusive	Positive	Useful—Positive	The evidence of uptake in PET confirms the presence of active inflammation
**2**	Inconclusive	Positive	Useful—Positive	The evidence of uptake in PET confirms the presence of active inflammation
**3**	Inconclusive	Negative	Useful—Negative	The lack of uptake in PET excludes the presence of active inflammation
**4**	Positive	Positive	Not useful—No reclassification	
**5**	Inconclusive	Negative	Useful—Negative	The lack of uptake in PET excludes the presence of active inflammation
**6**	Inconclusive	Negative	Useful—Negative	The lack of uptake in PET excludes the presence of active inflammation
**7**	Inconclusive	Negative	Useful—Negative	The lack of uptake in PET excludes the presence of active inflammation
**8**	Inconclusive	Positive	Useful—Positive	The evidence of uptake in PET confirms the presence of active inflammation
**9**	Inconclusive	Negative	Useful—Negative	The lack of uptake in PET excludes the presence of active inflammation
**10**	Inconclusive	Inconclusive	Not useful—No reclassification	
**11**	Inconclusive	Negative	Useful—Negative	The lack of uptake in PET excludes the presence of active inflammation
**12**	Negative	Negative	Not useful—No reclassification	

Abbreviations—MRI: magnetic resonance imaging; PET: positron emission tomography.

## Data Availability

The data presented in this study are available upon request from the corresponding author.

## Supplementary Materials

The following supporting information can be downloaded at: https://www.mdpi.com/article/10.3390/diagnostics15131670/s1.
